# Partial Resection of a Tailgut Cyst Attached to the Rectum via a Transcoccygeal Approach: A Case Report With a Favorable Outcome

**DOI:** 10.7759/cureus.80403

**Published:** 2025-03-11

**Authors:** Eitaro Okumura, Motoo Kubota, Ouji Momosaki, Ryo Hashimoto, Kotaro Kohara

**Affiliations:** 1 Spinal Surgery, Kameda Medical Center, Chiba, JPN

**Keywords:** colostomy creation, presacral space, rare benign tumor, tailgut cyst, transcoccygeal approach

## Abstract

A tailgut cyst is a congenital cystic tumor that develops when the tailgut, derived from the hindgut in early embryonic development, fails to naturally regress and remains in this presacral space. Treatment typically involves surgical resection due to the possibility of cyst infection and neurological symptoms from compression. Various surgical approaches exist, including abdominal, transanal, and transsacral approaches. We report a case where partial resection of a tailgut cyst attached to the rectum was performed via a transcoccygeal approach, resulting in a favorable outcome. The patient was a 20-year-old female with no significant medical history who had experienced constipation since middle school. At age 19, she developed urinary retention, and examination at a local clinic revealed a presacral cystic mass, leading to referral to our hospital. Upon presentation, she had constipation and urinary retention but no apparent neurological abnormalities. Blood tests were normal, and a pelvic MRI showed a 12 cm cystic mass posterior to the rectum. CT-guided aspiration revealed cloudy, yellowish, highly viscous fluid with no evidence of malignancy. Based on the fluid characteristics and imaging findings, a tailgut cyst was diagnosed. A transcoccygeal cyst resection was performed by transecting the bone distal to the sacral hiatus and mobilizing the coccyx. However, the tumor was adherent to the peritoneum and posterior rectal wall, resulting in partial resection. Pathology showed a cystic lesion lined with non-keratinizing stratified squamous epithelium and granulation tissue, with no malignant findings. Recurrence was noted two weeks postoperatively, and while complete resection was considered, the patient wished to avoid a colostomy. A second surgery using the same approach was performed, with maximal tumor dissection from peritoneal adhesions. Complete dissection from the posterior rectal wall was impossible, suggesting the tumor originated from this area. The remaining tumor was treated with holmium YAG (yttrium aluminum garnet) laser irradiation and anhydrous alcohol injection. MRI one month postoperatively showed complete cyst resolution, and after three years, there was no recurrence with good bowel and bladder function. We report a case of successful partial resection of a tailgut cyst attached to the rectum via a transcoccygeal approach. When the lesion invades the rectum, complete resection would necessitate a colostomy. However, in cases without malignant findings, choosing this minimally invasive approach with partial tumor resection and observation of the remaining rectal invasion site may be a viable option.

## Introduction

The retrorectal (presacral) space, surrounded by the anterior surface of the sacrum, posterior surface of the rectum, peritoneal reflection, and levator ani muscle, involves all three germ layers during embryonic development: the endoderm forming the digestive tract, the mesoderm forming the urogenital system, and the ectoderm forming the spinal cord and skin, leading to various congenital tumors [1]. A total of 60% of congenital presacral cysts are developmental cysts, which are further divided into epidermoid cysts, dermoid cysts, enterogenous cysts, teratomas, and finally, tailgut cysts [2]. The incidence of retrorectal lesions is low in adults. A ratio of around 1/40,000 patients has been reported based on the Mayo Clinic data [2]. A tailgut cyst develops from remnants of the hindgut during development [2]. During embryogenesis, the hindgut extends beyond the anal pit to reach the tail region, forming the tailgut [3]. Normally, this regresses by around eight weeks of gestation, but when it fails to regress and remains, it becomes a tailgut cyst [2,4]. It occurs predominantly in females with a ratio of 5:1 [5]. Tailgut cysts usually appear between the ages of 30 and 60 but can be present at any age [6-8]. Tailgut cyst presentation varies from asymptomatic incidental findings to symptomatic tumors due to pressure on organs and nerves [9]. The patients’ symptoms include lower back or perineal pain, constipation, urinary or fecal incontinence, urinary retention, rectal bleeding, and sexual or neurological dysfunctions. A history of recurrent anal sinus, fistulae, or abscesses is also an alarming sign [9]. Surgical resection is typically performed due to the risk of cyst infection and neurological symptoms from compression, with various approaches including abdominal, transanal, and transsacral methods. We report a case where partial resection of a tailgut cyst attached to the rectum was performed via a minimally invasive transcoccygeal approach, resulting in a favorable outcome.

## Case presentation

**Figure 1:**
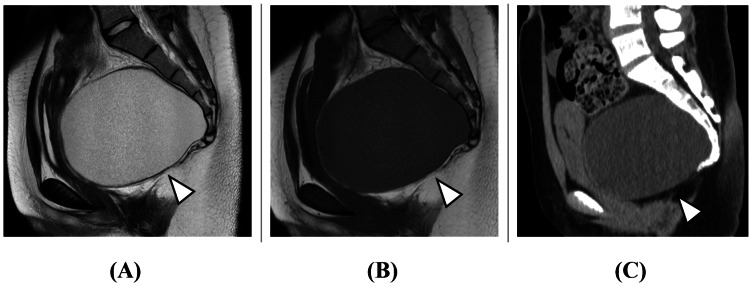
Initial pelvic imaging. (A) MRI T2 sagittal. (B) MRI T1 sagittal. (C) CT sagittal. A 12 cm cystic mass was observed posterior to the rectum, with homogeneous MRI T2 high intensity (arrowhead in Figure 1A), MRI T1 low intensity (arrowhead in Figure 1B), and CT iso density (arrowhead in Figure 1C) fluid content. The cyst wall was thin and uniform without apparent nodular lesions, suggesting against malignancy.

The patient was a 20-year-old female, independent with activities of daily living, with no significant medical history. She had experienced constipation since middle school, occasionally requiring enemas. At age 19, she developed urinary retention, and an ultrasound examination at a local urology clinic revealed a presacral cystic mass, and an ovarian tumor was suspected. However, after an MRI examination was performed, a tailgut cyst was suspected by the gynecology department. Subsequently, the patient was referred to a nearby gastrointestinal surgery department, where radical surgery via laparotomy was proposed. As the patient desired a less invasive surgical approach, she was referred to our hospital for consultation. Her presenting complaints were constipation and urinary retention. No apparent neurological abnormalities were observed. Blood tests, including complete blood count and biochemistry, were normal. A pelvic MRI showed a large cystic mass posterior to the rectum (Figure 1).

**Figure 2:**
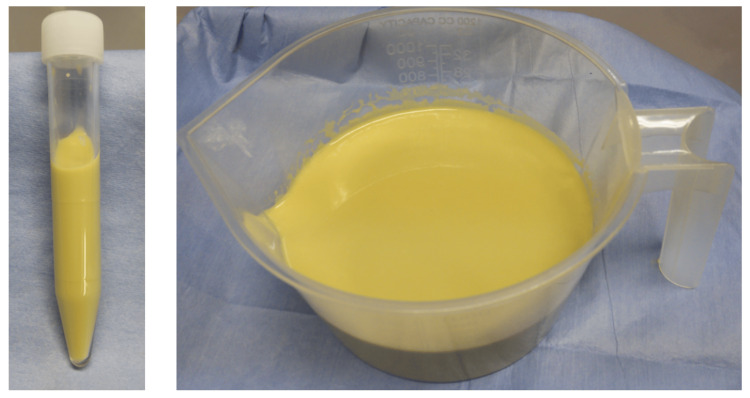
Aspiration fluid. The cyst contents were cloudy, yellowish, and highly viscous. Although numerous neutrophils were present, there were no bacteria, phagocytosis, or apparent cells.

While imaging findings did not suggest malignancy, CT-guided aspiration was performed for pathological examination (Figure 2).

**Figure 3:**
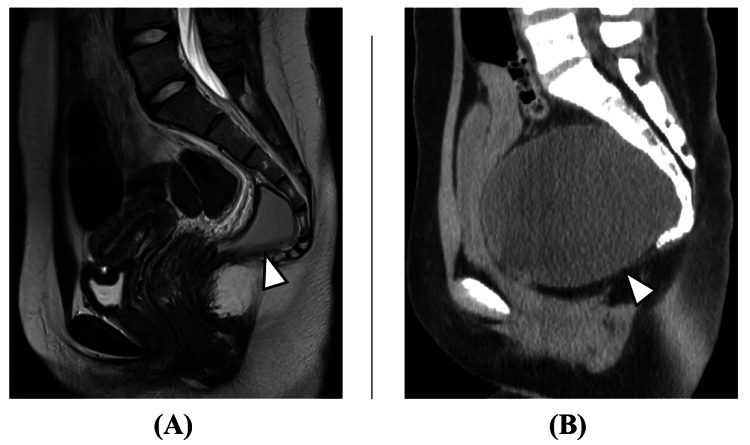
Preoperative pelvic imaging. (A) Immediately after aspiration (MRI T2 sagittal). (B) Three weeks after aspiration (CT sagittal). Approximately 500 ml of aspirated fluid had refilled within three weeks (arrowheads).

Although numerous neutrophils were present, there were no bacteria, phagocytosis, or apparent cells. The absence of glucose and lack of communication with cerebrospinal fluid led to the diagnosis of a tailgut cyst. Approximately 500 ml of aspirated fluid had been refilled within three weeks (Figure 3).

**Figure 4:**
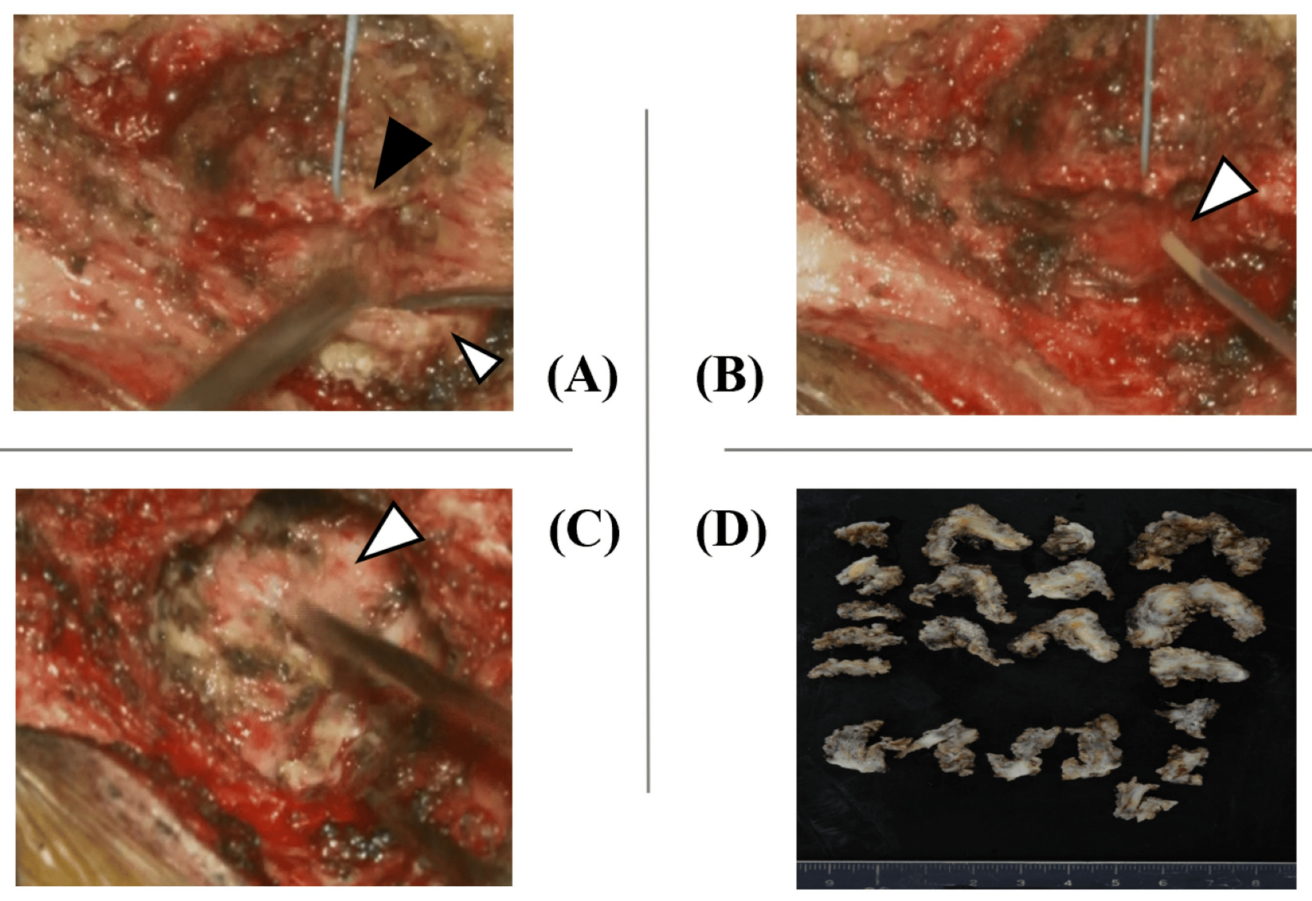
Initial surgery findings. The bone was transected distal to the sacral hiatus (black arrowhead in Figure 4A), and the coccyx (white arrowhead in Figure 4A) was mobilized. Intraoperative aspiration yielded yellowish fluid (arrowhead in Figure 4B). Maximum possible tumor resection was performed, but due to strong peritoneal adhesions, some tumor was left in place (arrowhead in Figure 4C). The tumor was resected in a piecemeal fashion due to its adhesions to surrounding tissues (D).

A transcoccygeal cyst resection was performed under general anesthesia in the Mohammedan position. A 7 cm skin incision was made from the coccygeal tip to the lower sacrum through the gluteal cleft. After dissecting from the lower sacrum to the coccyx, the sacral hiatus was identified. The bone was transected distal to the sacral hiatus, and the coccyx was mobilized. While dissecting the cyst wall with care to avoid soft tissue injury, the tumor was found to be circumferentially adherent. Particularly strong adhesions to the peritoneum suggested that complete tumor resection would risk peritoneal injury, so partial resection was performed (Figure 4).

**Figure 5:**
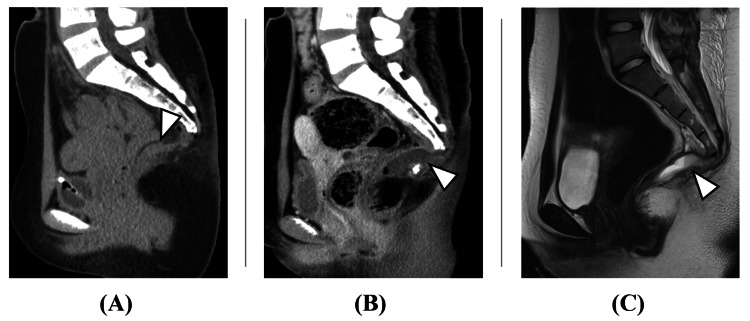
Post-initial surgery pelvic imaging. (A) Immediately post surgery (CT sagittal). (B) Two weeks post surgery (contrast CT sagittal). (C) Two weeks post surgery (MRI T2 sagittal). While immediate postoperative imaging showed cyst reduction with partial tumor remaining (arrowhead in Figure 5A), cyst fluid recurrence was observed two weeks after surgery (arrowheads in Figure 5B and Figure 5C).

Pathological examination of the resected specimen showed a cystic lesion lined with non-keratinizing stratified squamous epithelium and granulation tissue, with brain tissue and callus formation observed in the submucosa. The wall contained sebaceous tissue with foam cells and numerous lymphoplasmacytic infiltrates but no malignant findings. After the surgery, the symptoms were alleviated. While immediate postoperative imaging confirmed cyst reduction, drainage of fluid similar to the cyst contents began from the wound site on postoperative day 14, with imaging showing cyst recurrence (Figure 5).

**Figure 6:**
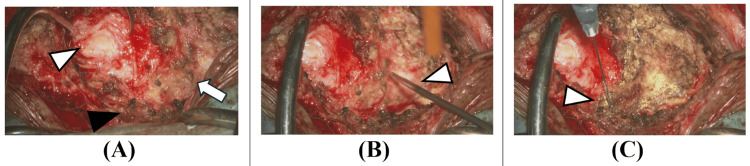
Reoperation findings. The tumor was resected until exposure of the retroperitoneum (white arrowhead in Figure 6A). The area where the tumor was attached to the rectum (black arrowhead in Figure 6A) was left in place without forced dissection (arrow in Figure 6A). The residual tumor was treated with holmium yttrium aluminum garnet (YAG) laser irradiation (arrowhead in Figure 6B), anhydrous alcohol injection (arrowhead in Figure 6C), and thorough cauterization.

The continuous drainage was thought to be secretions from the residual tumor. Although complete resection via reoperation was considered, the patient wished to avoid a colostomy, so a second surgery was performed using the same approach. While the tumor showed significant adhesion to the retroperitoneum, it was dissected and removed as much as possible. The area adherent to the posterior rectal wall could not be dissected, suggesting the tumor originated from the rectum. This portion of the tumor was left in place and thoroughly cauterized. Although there is no established evidence for their effectiveness in this tumor type, holmium YAG (yttrium aluminum garnet) laser irradiation and anhydrous alcohol injection were performed for residual tumor control (Figure 6).

**Figure 7:**
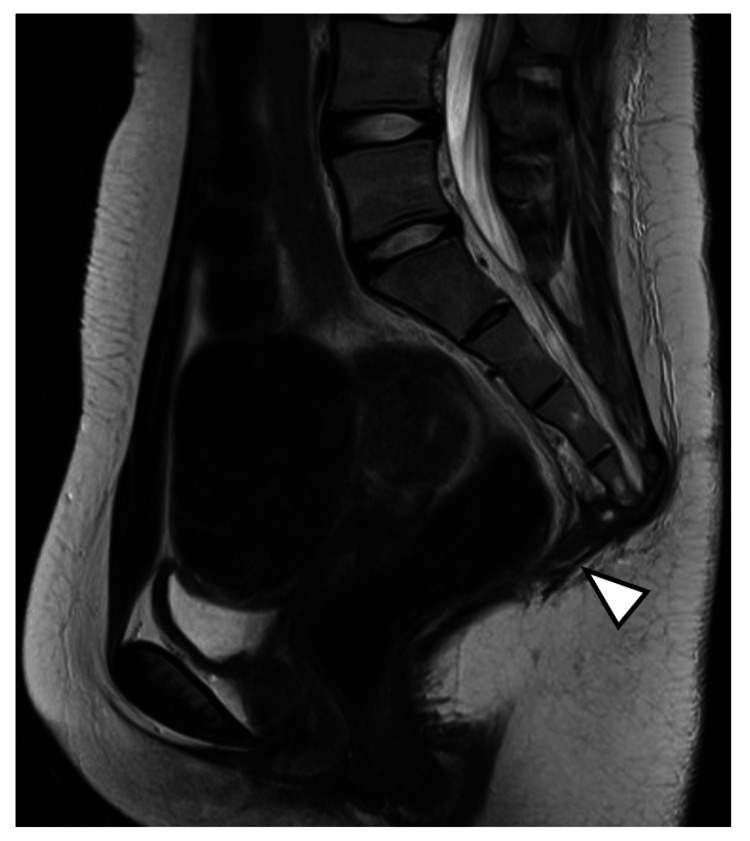
Post second surgery (MRI T2 sagittal). MRI one month postoperatively showed complete disappearance of the cyst (arrowhead).

The dead space after resection was filled with femoral fascia and adipose tissue. MRI one month postoperatively showed complete disappearance of the cyst (Figure 7).

There was no perineal sensory disturbance, and after three years of follow-up, there has been no tumor recurrence or enlargement, with normal bowel and bladder function.

## Discussion

Tailgut cyst presentations range from asymptomatic incidental findings to tumors causing organ or nerve compression symptoms. Specific symptoms can include low back pain, perineal pain, constipation, urinary incontinence, urinary retention, rectal bleeding, and sexual or neurological dysfunction [10]. Irregular or thickened cyst walls on CT or MRI suggest a malignancy risk. MRI accurately images soft tissues and can evaluate bone invasion and neural involvement [11]. While Mathis et al. recommend percutaneous presacral biopsy when malignancy is suspected [12], this carries a significant risk of dissemination in malignant lesions. Additionally, preoperative biopsy carries risks of infection and hematoma formation that may increase recurrence rates [13], making its use controversial. In our case, preoperative imaging showed uniform cyst walls without multilocularity or nodular lesions, suggesting against malignancy. However, as this information was crucial for determining the surgical approach, aspiration biopsy of the cyst contents was performed to confirm the absence of malignancy. For low-lying benign lesions not involving the rectum, the less invasive transsacral resection may be considered [14]. While transsacral resection has many advantages, including the absence of postoperative bowel adhesions and easier visualization of spinal canal relationships, abdominal or combined approaches are recommended when tumors extend above the S3 line or show rectal invasion [1,6,15]. Localio et al. recommend abdominal surgery or abdominoperineal resection for tumors larger than 8 cm or those with suspected infection or malignant changes [16]. Abdominal surgery allows reliable visualization of important intra-abdominal organs, making tumor resection more effective in cases of suspected malignancy. The transanal approach is selected for small, low-lying, uninfected lesions but theoretically carries a higher risk of pelvic infection as the rectum is used for access [6]. Mathis et al. reported that tailgut cyst resection can be safely and effectively performed primarily using posterior approaches [12]. In our case, although the tumor was large and extended above the S3 line, we chose the minimally invasive transcoccygeal approach. While complete resection is typically the goal as residual tumor can undergo malignant transformation to adenocarcinoma, carcinoid, or neuroendocrine carcinoma [17,18], intraoperative findings revealed continuity between the cyst and posterior rectal wall, with the lesion palpable through the posterior rectal wall on digital examination. Complete tumor resection would have necessitated a colostomy. Considering the patient's young age and desire to avoid colostomy, only the tumor on the posterior rectal surface was left in place. Regarding holmium YAG laser irradiation and anhydrous alcohol injection for the tumor, while holmium YAG laser enables tissue vaporization and coagulation in urinary tract tumor surgery [19], and anhydrous ethanol injection utilizes protein coagulation effects in liver cancer treatment [20], neither has established efficacy for this tumor type. Therefore, their contribution to residual tumor control in this case remains unclear. While the risk of malignant transformation in residual tumor was previously estimated at 2% [10], Mathis et al. reported 13% [12]. Our patient shows no tumor recurrence or enlargement after three years, with good bowel and bladder function. Continued careful monitoring for residual lesion enlargement or malignant transformation is necessary.

## Conclusions

We report a case of successful partial resection of a tailgut cyst attached to the rectum via a transcoccygeal approach. The transcoccygeal approach provides relatively easy access to the presacral region with minimal invasiveness and avoids entering the peritoneal cavity. While complete tumor resection is the basic treatment strategy, when the lesion involves the rectum, complete resection would necessitate colostomy. However, in cases without malignant findings, choosing this approach with maximal tumor resection while leaving the rectal invasion site and continuing observation may be a viable option.
